# Telemedicine Usage Among Urologists During the COVID-19 Pandemic: Cross-Sectional Study

**DOI:** 10.2196/21875

**Published:** 2020-11-05

**Authors:** Justin M Dubin, W Austin Wyant, Navin C Balaji, William LK Ong, Reda H Kettache, Malik Haffaf, Skander Zouari, Diego Santillan, Ana Maria Autrán Gómez, Hossein Sadeghi-Nejad, Stacy Loeb, James F Borin, Juan Gomez Rivas, Jeremy Grummet, Ranjith Ramasamy, Jeremy Y C Teoh

**Affiliations:** 1 Department of Urology University of Miami Miller School of Medicine Miami, FL United States; 2 Department of Urology Penang General Hospital Penang Malaysia; 3 Department of Urology Bachir Bennacer - Biskra Hospital Biskra Algeria; 4 Department of Urology EHU 1er Novembre Oran Algeria; 5 Urology Department Charles Nicolle Hospital Tunis Tunisia; 6 Department of Urology Hospital Italiano de Buenos Aires Buenos Aires Argentina; 7 Department of Urology Hospital Universitario Fundación Jiménez Diaz Madrid Spain; 8 Research Office of Confederacion Americana de Urologia Buenos Aires Argentina; 9 Department of Urology Rutgers New Jersey Medical School Newark, NJ United States; 10 Department of Urology Hackensack Meridian Health Hackensack, NJ United States; 11 Department of Urology and Population Health New York University and Manhattan Veterans Affairs Medical Center New York City, NY United States; 12 Department of Urology New York University New York City, NY United States; 13 Department of Urology La Paz University Hospital Madrid Spain; 14 Department of Urology Autonomous University of Madrid Madrid Spain; 15 Department of Surgery Central Clinical School Monash University Melbourne Australia; 16 SH Ho Urology Centre, Prince of Wales Hospital Department of Surgery The Chinese University of Hong Kong Hong Kong China

**Keywords:** coronavirus, COVID-19, technology, telemedicine, telehealth, urology, cross-sectional, perception, usability, barrier, implementation

## Abstract

**Background:**

Prior to the COVID-19 pandemic, urology was one of the specialties with the lowest rates of telemedicine and videoconferencing use. Common barriers to the implementation of telemedicine included a lack of technological literacy, concerns with reimbursement, and resistance to changes in the workplace. In response to the COVID-19 pandemic declared in March 2020, the delivery of urological services globally has quickly shifted to telemedicine to account for the mass clinical, procedural, and operative cancellations, inadequate personal protective equipment, and shortage of personnel.

**Objective:**

The aim of this study was to investigate current telemedicine usage by urologists, urologists’ perceptions on the necessity of in-person clinic appointments, the usability of telemedicine, and the current barriers to its implementation.

**Methods:**

We conducted a global, cross-sectional, web-based survey to investigate the use of telemedicine before and after the COVID-19 pandemic. Urologists’ perceived usability of telemedicine was assessed using a modified Delphi approach to create questions based on a modified version of the validated Telehealth Usability Questionnaire (TUQ). For the purposes of this study, telemedicine was defined as video calls only.

**Results:**

A total of 620 urologists from 58 different countries and 6 continents participated in the survey. Prior to COVID-19, 15.8% (n=98) of urologists surveyed were using telemedicine in their clinical practices; during the pandemic, that proportion increased to 46.1% (n=283). Of the urologists without telemedicine experience, interest in telemedicine usage increased from 43.7% (n=139) to 80.8% (n=257) during the COVID-19 pandemic. Among urologists that used telemedicine during the pandemic, 80.9% (n=244) were interested in continuing to use it in their practice. The three most commonly used platforms were Zoom, Doxy.me, and Epic, and the top three barriers to implementing telemedicine were patients’ lack of technological comprehension, patients’ lack of access to the required technology, and reimbursement concerns.

**Conclusions:**

This is the first study to quantify the use, usability, and pervading interest in telemedicine among urologists during the COVID-19 pandemic. In the face of this pandemic, urologists’ usage of telemedicine nearly tripled, demonstrating their ability to adopt and adapt telemedicine into their practices, but barriers involving the technology itself are still preventing many from utilizing it despite increasing interest.

## Introduction

According to the American Telemedicine Association, telemedicine is the “use of medical information exchanged from one site to another via electronic communications to improve a patient’s clinical health status” [1]. Telehealth, in comparison, is a combination of both telemedicine and remote nonclinical services such as provider training, administrative meetings, and continuing medical education. A 2016 survey by the American Medical Association [2] found that although most medical specialties use some form of telemedicine, videoconferencing is most used by physicians in emergency medicine, psychiatry, and pathology. Urology was found to be one of the specialties with the lowest rates of telemedicine and videoconferencing use. In a recent review, urologists commonly cited a lack of technological literacy, concerns with reimbursement, and resistance to changes in the workplace as barriers to the implementation of telemedicine in their practices [3].

In response to the COVID-19 pandemic declared in March 2020, the delivery of urological services globally has quickly shifted to account for the unprecedented mass clinical, procedural, and operative delays/cancellations, inadequate personal protective equipment, and shortage of personnel [4,5]. To accommodate the limited access to operative rooms, the surgical and urological communities took action: the American College of Surgeons recommended against elective surgeries and a multidepartmental group of urologists published urological surgery triage guidelines [6,7]. Globally, health care systems aggressively pushed for increased usage of telemedicine, with institutions such as New York University increasing their telemedicine use by over 4000% during this time [8]. The increase in telemedicine has inspired several organizations, including the European Association of Urology, to create recommendations for its professional usage, and some subspecialties have even provided guidelines for treating specific conditions via telemedicine [9,10]. The major shift in health care brought on by the COVID-19 pandemic has also seemingly incentivized all health care practices to more readily adopt telemedicine in their practices. The pandemic has exacerbated health inequities on a global scale, and we wanted to investigate whether urologists and their practices were able to adapt to and overcome the previous barriers preventing them from prior telemedicine use. To our knowledge, however, the actual increase in urologists’ usage of telemedicine during the pandemic has not been quantified. We hypothesized that, in response to the pandemic, urologists’ use of telemedicine has increased. Our objective was to examine how urologists perceive the necessity of in-person clinic appointments, the usability of telemedicine, and the current barriers to its implementation.

## Methods

We conducted a global, cross-sectional, web-based survey to investigate the use of telemedicine before and after the COVID-19 pandemic. Urologists’ perceived usability of telemedicine was assessed using a modified Delphi approach to create questions based on a modified version of the validated Telehealth Usability Questionnaire (TUQ) [11]. The TUQ is a validated survey that was designed to assess both clinician and patient usability of telehealth implementation and services and previous studies have utilized it to evaluate telemedicine from the perspective of both the patient and the provider [12,13]. For the purposes of this study, telemedicine was defined as video calls only.

From April 15 to May 9, 2020, we distributed a 41-item survey to practicing urologists by email, via individual institutions, professional urology organizations, and Twitter. Distribution of the survey included an institutional review board (IRB)–approved introduction and invitation to complete the survey with a link that sent the user to the Qualtrics website to complete our survey. The survey was distributed via email and on Twitter. The survey included questions on urologist demographics, experiences with telemedicine prior to COVID-19, experiences with telemedicine during COVID-19, interest in telemedicine usage, barriers to telemedicine usage, and the TUQ to assess the telemedicine platform the provider was using. The survey was completely anonymous and took no longer than 8 minutes to complete. The professional organizations surveyed included the New York, Northeastern, North Central, South Central, and Southeastern sections of the American Urological Association; the European Society of Residents in Urology; the European Association of Urology Section of Uro-Technology; Young Academic Urologists; the Spanish Urology Residents Working Group; the Sexual Medicine Society of North America; the Urological Society of Australia and New Zealand; and the Endourology Society. This project was approved by the IRB of the University of Miami (reference number: 20200414; approved 4/9/2020).

Statistical analysis was done using Microsoft Excel (Microsoft Corp) and MATLAB (version R2020a, The Mathworks Inc). We sought to compare our modified TUQ scores for the top three telehealth platforms used, as indicated by the survey results. The modified TUQ score was broken up into 5 categories for assessing telehealth platforms: usefulness, ease of use and learnability, interface quality, interaction quality, and reliability, with each question being answered based on a 7-point Likert scale. To calculate TUQ scores for each category, responses to all of the questions within each subsection of an individual’s survey were averaged. Each category subsection was composed of 3 or 4 questions, based on the respective category. Next, all of the respondent’s subsection scores, sorted by the telehealth platform used, were averaged to generate an overall subsection score stratified by platform used. These scores were compared using a single-factor ANOVA (analysis of variance), with an alpha of .05. Chi-squared tests were performed, with an alpha of .05. These results are expressed as total occurrences and corresponding percentages.

## Results

### Demographics

A total of 676 urologists clicked into the survey; however, after removing 56 incomplete surveys that were deemed to have insufficient data to be included, the final data set evaluated a total of 620 urologists from 58 different countries and 6 continents who participated in the survey. Of the 620 included, 24 were incomplete but were considered to have sufficient data to be included in the final data set. By continent, participation included 340 urologists from North America, 102 urologists from Europe, 65 urologists from Asia, 63 urologists from South America, 25 urologists from Africa, and 25 urologists from Australia. The three countries with the most participants were the United States (n=311, 50%), Spain (n=43, 6.9%), and Argentina (n=34, 5.5%). Further general demographics can be seen in Table 1. When evaluating participating urologist practices in terms of subspecialization, we divided practices into 4 groups: general (n=156, 25.2%), oncology (n=259, 41.8%), pediatrics (n=42, 6.8%), and nononcologists (n=163, 26.3%). Data on survey responses based on this subspecialty grouping are provided in Table 2. Overall, there were no significant differences in perceptions or usage of telemedicine among the subspecialty groups.

**Table 1:** Demographic data.

Variable		Respondents (N=620), n (%)
**Gender**		
	Male	512 (82.6)
	Female	101 (16.3)
	Other	2 (0.3)
	Prefer not to answer	5 (0.8)
**Age (years)**		
	<30	23 (3.7)
	30-39	183 (29.5)
	40-49	183 (29.5)
	50-59	129 (20.8)
	≥60	102 (16.5)
**Years of experience**		
	<5	144 (23.2)
	5-10	119 (19.2)
	11-15	98 (15.8)
	16-20	73 (11.8)
	>20	186 (30.0)
**Subspecialty**		
	General	156 (25.2)
	Oncology	259 (41.8)
	Pediatrics	42 (6.8)
	Nononcology	163 (26.3)
**Continent**		
	Africa	25 (4.0)
	Asia	65 (10.5)
	Australia	25 (4.0)
	Europe	102 (16.5)
	North America	340 (54.8)

**Table 2:** Subspecialty grouped data.

Variable		Total, n (%)	General, n (%)	Oncology, n (%)	Pediatrics, n (%)	Nononcologists, n (%)	*P* value^a^
**Clinic-anticipated inpatient consultation need**							.49
	<25%	144 (23.2)	33 (21.3)	59 (22.8)	9 (21.4)	43 (26.4)	
	25%-49%	119 (19.2)	31 (19.9)	55 (21.2)	10(23.8)	23 (14.1)	
	50%	98 (15.8)	23 (14.7)	41 (15.8)	10 (23.8)	24 (14.7)	
	51%-75%	73 (11.8)	14 (9.0)	32 (12.4)	5 (11.9)	22 (13.5)	
	>75%	186 (30)	55 (35.3)	72 (27.8)	8 (19.0)	51 (31.3)	
Surrogate use of telemedicine before COVID-19		302 (100)	22 (27.2)	43 (35.8)	9 (37.5)	24 (31.2)	.57
Have you ever used telemedicine?		620 (100)	81 (51.9)	121 (46.7)	24 (57.1)	76 (46.6)	.47
**Clinic appointment using telemedicine prior** **to COVID-19**							.57
	<25%	83 (84.7)	17 (77.3)	36 (83.7)	8 (88.9)	22 (91.7)	
	25%-49%	7 (7.1)	2 (9.1)	4 (9.3)	0 (0)	1 (4.2)	
	50%	4 (4.1)	2 (9.1)	2 (4.7)	0 (0)	0 (0)	
	51%-75%	1 (1.0)	1 (4.5)	0 (0)	0 (0)	0 (0)	
	>75%	3 (3.1)	0 (0)	1 (2.3)	1 (11.1)	1 (4.2)	
**Interest in using telemedicine for clinic appointments before COVID-19**							.11
	Not at all interested	65 (20.5)	23 (30.7)	20 (14.4)	2 (11.1)	20 (23.5)	
	Not very interested	72 (22.7)	17 (22.7)	37 (26.6)	6 (33.3)	12 (14.1)	
	Neutral	42 (13.2)	7 (9.3)	22 (15.8)	3 (16.7)	10 (11.8)	
	Somewhat interested	86 (27.1)	19 (25.3)	34 (24.5)	6 (33.3)	27 (31.8)	
	Very interested	52 (16.4)	9 (12)	26 (18.7)	1 (5.6)	16 (18.8)	
Surrogate use telemedicine since COVID-19		301 (100)	76 (93.8)	113 (95)	24 (100)	73 (94.8)	.68
**Percentage of conversion to telemedicine since COVID-19**							.39
	<25%	86 (30.1)	25 (32.9)	36 (31.9)	10 (41.7)	15 (20.5)	
	25%-49%	57 (19.9)	20 (26.3)	21 (18.6)	3 ( 12.5)	13 (17.8)	
	50%	42 (14.7)	8 (10.5)	18 (15.9)	4 (16.7)	12 (16.4)	
	51%-75%	34 (11.9)	9 (11.8)	12 (10.6)	4 (16.7)	9 (12.3)	
	>75%	67 (23.4)	14 (18.4)	26 (23.0)	3 (12.5)	24 (32.9)	
**Interest to continue telemedicine as surrogate after experience**							.497
	Not at all interested	11 (3.9)	4 (5.3)	2 (1.8)	1 (4.2)	4 (5.6)	
	Not very interested	25 (8.8)	7 (9.2)	12 (10.8)	1 (4.2)	5 (6.9)	
	Neutral	18 (6.4)	5 (6.6)	7 (6.3)	3 (12.5)	3 (4.2)	
	Somewhat interested	82 (29)	29 (38.2)	27 (24.3)	7 (29.2)	19 (26.4)	
	Very interested	147 (51.9)	31 (40.8)	63 (56.8)	12 (50.0)	41 (56.9)	
Telemedicine for interaction with hospital inpatients prior to COVID-19		37 (12.4)	13 (16.0)	15 (12.8)	2 (8.3)	7 (9.2)	.001
Telemedicine for interaction with hospital inpatient since COVID-19		55 (19.4)	16 (20.5)	24 (20.7)	7 (29.2)	8 (12.3)	.29
**Percentage of conversion to telemedicine for inpatient visits**							.27
	<25%	19 (35.2)	9 (56.3)	8 (33.3)	0 (0)	2 (25)	
	25%-49%	11 (20.4)	2 (12.5)	5 (20.8)	3 (50)	1 (12.5)	
	50%	7 (13.0)	2(12.5)	2 (8.3)	2 (33.3)	1(12.5)	
	51%-75%	8 (14.8)	2 (12.5)	4 (16.7)	1 (16.7)	1 (12.5)	
	>75%	9 (16.7)	1 (6.3)	5 (20.8)	0 (0)	3 (37.5)	
**Interested in using telemedicine before?**							.89
	Not at all interested	25 (11.4)	7 (11.7)	8 (9.1)	2 (11.8)	8 (14.8)	
	Not very interested	37 (16.9)	13 (21.7)	12(13.6)	4 (23.5)	8 (14.8)	
	Neutral	41 (18.7)	12 (20.0)	18 (20.5)	2 (11.8)	9 (16.7)	
	Somewhat interested	66 (30.1)	14 (23.3)	28 (31.8)	7 (41.2)	17 (31.5)	
	Very interested	50 (22.8)	14 (23.3)	22 (25.0)	2 (11.8)	12 (22.2)	
**Interested in using telemedicine after experience**							.45
	Not at all interested	3 (5.6)	0 (0)	3 (12.5)	0 (0)	0 (0)	
	Not very interested	2 (3.7)	1 (6.3)	1 (4.2)	0 (0)	0 (0)	
	Neutral	9 (16.7)	4 (25.0)	2 (8.3)	0 (0)	3 (37.5)	
	Somewhat interested	20 (37.0)	7 (43.8)	9 (37.5)	2 (33.3)	2(25.0)	
	Very interested	20 (37.0)	4 (25.0)	9 (37.5)	4 (66.7)	3 (37.5)	
**Location of telemedicine**							.18
	At home	108 (38.2)	27 (35.1)	36 (32.4)	12 (52.2)	33 (45.8)	
	In office	169 (59.7)	50 (64.9)	71 (64)	11 (47.8)	37 (51.4)	
	At other places	6 (2.1)	0 (0)	4 (3.6)	0 (0)	2 (2.8)	
**Interested in interacting with future patients with telemedicine after experience**							.01
	Not at all interested	7 (2.2)	3 (4)	2 (1.5)	0 (0)	2 (2.4)	
	Not very interested	12 (3.8)	4 (5.3)	4 (3)	2 (11.1)	2 (2.4)	
	Neutral	41 (13.1)	9 (12)	20 (14.9)	0 (0)	12 (14.1)	
	Somewhat interested	122 (39.1)	36 (48)	58 (43.3)	9 (50)	19 (22.4)	
	Very interested	130 (41.7)	23 (30.7)	50 (37.3)	7 (38.9)	50 (58.8)	

^a^Chi-squared test.

### Barriers to Telemedicine Use

Approximately half (n=318, 51.2%) of the urologists surveyed have never used telemedicine. When assessing the barriers to telemedicine use, the top three reasons urologists gave were patients’ lack of technological comprehension, patients’ lack of access to required technology, and reimbursement concerns (Table 3). Another barrier of significance was lack of administrative support, which was the fourth most frequently mentioned barrier to telemedicine use. Of note, the question regarding barriers to usage had a “check all that apply” option, which explains why the percent of cases exceeds 100% in Table 3.

**Table 3:** Barriers to telemedicine use.

Reasons	Responses, n (%)	Cases (%)
Patients lack technological comprehension to use it	183 (18.8)	58.3
Patients lack access to necessary technology	174 (17.9)	55.4
Insurance reimbursement concerns	163 (16.7)	51.9
Lack of administrative support	142 (14.6)	45.2
Legal concerns	118 (12.1)	37.6
Practice lacks technology for telemedicine	118 (12.1)	37.6
Practice lacks finances for telemedicine	54 (5.5)	17.2
Other	22 (2.3)	7.0
Total	974 (100.0)	310.2

### Telemedicine Use During the COVID-19 Pandemic

Prior to COVID-19, only 15.8% (n=98) of urologists surveyed were using telemedicine in their clinical practices. During the pandemic, however, 46.1% (n=283) of all urologists surveyed were using telemedicine in their clinical practices. Since the start of COVID-19, 50% (n=143) of participating urologists converted at least half of their originally scheduled in-person clinic visits to telemedicine. Despite this increase in usage, 68% (n=421) of all urologists surveyed believed that at least half of their clinic appointments required an in-person visit. Urologists with prior telemedicine experience were less likely to believe that 50% of their patients required in-person visits when compared to those without telemedicine experience (43.0% [n=130] vs 52.8% [n=168], *P*=.015). In the inpatient setting, 6% (n=37) of surveyed urologists had used telemedicine prior to COVID-19 to interact with urology hospital inpatients. During the pandemic, 8.9% (n=55) of participating urologists utilized telemedicine in the inpatient setting.

Among the participating urologists without telemedicine experience, interest in usage of telemedicine increased from 43.7% (n=139) to 80.8% (n=257) during COVID-19. After using telemedicine during the pandemic, 80.9% (n=244) of urologists surveyed were interested in continuing to use it in their practice. About half of participating urologists (n=116, 52.9%) were interested in utilizing telemedicine in the inpatient hospital setting. The majority of sampled urologists who had experienced inpatient telemedicine use during the pandemic were interested in continuing its usage (n=308, 74%), and about half (n=29, 53%) of the urologists who had not used it in that setting were interested in doing so.

### Assessment of Telemedicine Platforms

The 5 most common platforms used by participating urologists for telemedicine visits were Zoom, Doxy.me, Epic, WhatsApp, and Skype. Telemedicine was mostly done either in the office (n=169, 59.7%) or at home (n=108, 38.2%). We compared the scores of the 3 most commonly used platforms (Zoom, Doxy.me, Epic) by usefulness, ease of use and learnability, interface quality, interaction quality, and reliability. There were no significant differences between the platforms in any category (Table 4).

**Table 4:** Usability scores of different telemedicine platforms.

Score and platform		Mean (SD)	F	F_critical_	*df*	*P* value
**Usefulness**			1.82	3.10	2	.17
	Overall	5.05 (1.24)				
	Zoom	4.89 (1.31)				
	Doxy.me	4.95 (1.28)				
	Epic	5.67 (0.57)				
**Ease of use and learnability**			2.12	3.10	2	.12
	Overall	5.46 (1.20)				
	Zoom	5.51 (1.00)				
	Doxy.me	5.04 (1.31)				
	Epic	5.61 (0.76)				
**Interface quality**			0.03	3.10	2	.97
	Overall	4.98 (1.17)				
	Zoom	4.99 (1.11)				
	Doxy.me	4.93 (1.23)				
	Epic	4.93 (0.93)				
**Interaction quality**			0.86	3.10	2	.43
	Overall	5.04 (1.14)				
	Zoom	5.07 (1.11)				
	Doxy.me	4.74 (1.26)				
	Epic	5.07 (0.98)				
**Reliability**			0.76	3.10	2	.47
	Overall	3.93 (1.16)				
	Zoom	3.95 (1.25)				
	Doxy.me	3.63 (1.17)				
	Epic	3.73 (1.05)				

Assessment of individual TUQ questions provided insight into participating urologists’ attitudes toward telemedicine use (Table 5). The majority of the urologists using telemedicine agreed that it improves patient access (n=223, 78.5%), saves physician travel time to the hospital (n=168, 55.6%), and addresses patients’ health care needs (n=229, 80.6%). Evaluating ease of use, the majority of surveyed urologists found telemedicine simple to use (n=220, 77.5%), easy to learn (n=253, 89.1%), and felt they could be productive using it (n=213, 75%). Overall, 75.4% (n=214) of sampled urologists said they liked using telemedicine, but 57% (n=162) felt that telemedicine visits were not the same as in-person visits and 47.6% (n=135) did not think they could see their patients as well as if they were in person.

**Table 5:** Telehealth Usability Questionnaire survey data (N=284 responses).

Question	Strongly disagree, n (%)	Disagree, n (%)	Somewhat disagree, n (%)	Neutral, n (%)	Somewhat agree, n (%)	Agree, n (%)	Strongly agree, n (%)
Q25. TM^a^ improves patient access to me	10 (3.5)	11 (3.9)	9 (3.2)	31 (10.9)	66 (23.2)	96 (33.8)	61 (21.5)
Q26. TM saves me traveling time to the hospital or clinic	18 (6.3)	47 (16.5)	11 (3.9)	50 (17.6)	44 (15.5)	71 (25)	43 (15.1)
Q27. TM provides for patient health care needs	6 (2.1)	10 (3.5)	20 (7.0)	19 (6.7)	82 (28.9)	110 (38.7)	37 (13)
Q28. TM was simple to use	8 (2.8)	14 (4.9)	25 (8.8)	17 (6.0 )	49 (17.3)	113 (39.8)	58 (20.4)
Q29. TM was easy to learn to use	4 (1.4)	4 (1.4)	8 (2.8)	15 (5.3)	38 (13.4)	137 (48.2)	78 (27.5)
Q30. I could become productive quickly using TM	6 (2.1)	14 (4.9)	19 (6.7)	32 (11.3)	58 (20.4)	101 (35.6)	54 (19)
Q31. Interaction with TM is pleasant	7 (2.5)	7 (2.5)	17 (6)	26 (9.2)	71 (25)	115 (40.5)	41 (14.1)
Q32. I like using TM	6 (2.1)	13 (4.6)	26 (9.2)	25 (8.8)	57 (20.1)	109 (38.4)	48 (16.9)
Q33. TM is simple and easy to understand	4 (1.4)	7 (2.5)	4 (1.4)	25 (8.8)	54 (19)	141 (49.6)	49 (17.3)
Q34. TM is able to do everything I want it to do	31 (10.9)	52 (18.3)	55 (19.4)	26 (9.2)	62 (21.8)	42 (14.8)	16 (5.6)
Q35. I can easily talk with patients on TM	1 (0.4)	11 (3.9)	19 (6.7)	29 (10.2)	63 (22.2)	110 (38.7)	51 (18)
Q36. I can hear patient easily on TM	2 (0.7)	9 (3.2)	18 (6.3)	28 (9.9)	68 (23.9)	113 (39.8)	46 (16.2)
Q37. I was able to express myself effectively on TM	1 (0.4)	10 (3.5)	15 (5.3)	19 (6.7)	62 (21.8)	130 (45.8)	47 (16.5)
Q38. I can see the patient as well as if we met in person	24 (8.5)	49 (17.3)	62 (21.8)	25 (8.8)	59 (20.8)	44 (15.5)	21 (7.4)
Q39. Visits over TM are the same as in-person visits	44 (15.5)	73 (25.7)	45 (15.8)	25 (8.8)	59 (20.8)	24 (8.5)	14 (4.9)
Q40. I can recover quickly and easily from mistakes I made using TM	3 (1.1)	14 (4.9)	21 (7.4)	80 (8.2)	57 (20.1)	81 (28.5)	28 (9.9)
Q41. TM gave error signals that told me how to fix the problem.	23 (8.1)	68 (23.9)	25 (8.8)	105 (37)	34 (12)	24 (8.5)	5 (1.8)

^a^TM: telemedicine.

## Discussion

### Principal Findings

The COVID-19 pandemic has resulted in policies that have limited direct human interactions on a global scale. These limitations have drastically modified social and professional practices, especially within medicine. In an attempt to reduce transmission of COVID-19, there has been a large push for expansion of telemedicine usage throughout the medical field, including within urology. We wanted to explore how practicing urologists are adapting to using telemedicine during COVID-19, the usability of the telemedicine platforms, and the potential barriers to its use. Our results indicate that telemedicine was readily adopted during the pandemic as its usage among surveyed urologists almost tripled from 15.8% to 46.1%. Experiences during COVID-19 dramatically increased interest in telemedicine use among those participating urologists without access to telemedicine (43.7% to 80.8%), and 80.9% of participating urologists using telemedicine were interested in continuing its usage. Despite the increased usage and interest in the continued usage of telemedicine during COVID-19, approximately half (51.2%) of the urologists surveyed had never used telemedicine, and the majority of urologists still believed that at least half of their clinic appointments require an in-person visit.

Prior to the pandemic, few urologists were using telemedicine due to lack of technological literacy, concerns over reimbursement, and resistance to changes in the workplace [3]. Our data confirms that the majority of urological practices were not utilizing telemedicine prior to the pandemic, as only 15.8% of sampled urologists were employing it in their practices. Despite the push for increased use of telemedicine during the pandemic, it is concerning that approximately half of participating urologists were still not using telemedicine. Evaluating the top three barriers for telemedicine use, the main obstacle appears to be the technology itself. Technological issues, including disparities in internet access and poor audio quality in patients who speak non-English languages, have been described as barriers to telemedicine care during the pandemic [14]. A systematic review assessing the barriers to telemedicine adoption worldwide also confirmed that technology-specific issues were the main barriers to telemedicine use, with the most frequently cited barrier as technically challenged staff [15]. One possible way to help overcome some of these barriers could be through novel approaches using social media. Twitter-based journal clubs have been shown to be efficient means of disseminating information in a free and time-efficient manner [16]. Twitter-based educational strategies that educate health care providers, administrators, and patients on telehealth could facilitate previous apprehensions with the use of the technology and promote further usage. As telemedicine continues to play a major role in medical practices, hopefully health care systems and providers will invest more time and effort into educating patients on and providing better access to telemedicine.

Although only half of surveyed urologists were using telemedicine during the pandemic, those urologists with experience in telemedicine successfully incorporated it into their practices during quarantine. Health care systems were able to accommodate an increased telemedicine load since approximately 50% of participating urologists converted half of their originally scheduled in-person clinic visits to remote ones. A systematic review of telehealth in urology showed that telehealth can be successfully implemented in urology patients with prostate cancer, urinary incontinence, pelvic organ prolapse, uncomplicated urinary stones, and uncomplicated urinary tract infections, but the pandemic has forced urologists to assimilate virtually all urological conditions to telemedicine [17]. This rapid pivot to telemedicine in the outpatient setting is encouraging, demonstrating that urologists, patients, and health care systems alike are willing to provide and accept care over telemedicine. The prompt assimilation to telemedicine during the pandemic was well documented by the urology department at the tertiary academic center Charleston Area Medical Center. They demonstrated that quick telemedicine adoption was feasible; by day 5 of their transition, more patients were participating in audiovisual video encounters than any other modality. Just as important, no office staff found the introduction of telemedicine stressful, and more than 80% of telemedicine patients were interested in future encounters [18]. Despite these successes, our data still showed that lack of administrative support was a major barrier for many urologists to adopt telemedicine into their practice. With almost 15% of participating urologists citing it as a barrier, we need to acknowledge that there are overarching health system issues, not just patient and physician issues, that are preventing many from gaining access to telemedicine.

The success of transitioning to telemedicine is also dependent on patient acceptance of the technology. Data prior to COVID-19 already suggested patients are embracing telemedicine, but in the face of the pandemic, desire for telemedicine consultations as a replacement for in-person visits among urology patients was very high [19,20]. There are concerns about telemedicine and how it may introduce additional stressors to already vulnerable patients, especially the elderly who may lack technical knowledge or have disabilities such as hearing loss. In these cases, it is up to the urologist to accommodate these patients accordingly and ensure through their best practices that patients are engaging in clearly communicated encounters that includes shared decision making [21]. Although there is still significant room for improvement, the successful transition to telemedicine during the pandemic demonstrated health care systems can change rapidly and effectively when there is consensus between physicians, health care administrators, and patients.

Overall, telemedicine experiences during COVID-19 appear to have changed urologists’ interest in future telemedicine usage. Urologists’ experiences with telemedicine during the pandemic appears to have been positive with most participating urologists (80.9%) now interested in continuing to incorporate it in their practices. The majority of surveyed urologists using telemedicine agreed that it improves patient access (78.5%), saves physician travel time to the hospital (55.6%), and provides patients with their health care needs (80.6%). Most participants found telemedicine simple to use (77.5%), easy to learn (89.1%), and felt they could be productive using it (75%). Overall, 75.4% of urologists said they liked using telemedicine. The pandemic demonstrated the many benefits of telemedicine, which include reduced spread of COVID-19, reduced contamination of uninfected persons, reduced transmission to hospital workers and hospital surfaces, increased appointment convenience for patients, increased patient satisfaction, and provided physicians the opportunity to work from home [9]. Even though the majority of surveyed urologists wanted to incorporate telemedicine in their practice, 68% of participating urologists still believed that at least half of their clinic appointments required an in-person visit. Subanalysis of this population, however, demonstrated more acceptance of telemedicine as a replacement for in-person visits among those urologists using telemedicine. Urologists with prior telemedicine experience were less likely to believe that 50% of their patients required in-person visits when compared to those without telemedicine experience. Grouping participating urologists into general, oncology, pediatrics, and nononcology groups showed no significant differences in telemedicine practices or opinions. Our data suggest that even though participating urologists have experienced success with telemedicine, they still believed that most patients benefit from in-person visits. Further studies are needed to assess the advantages and disadvantages of telemedicine compared to in-person visits.

A major factor in the success of telemedicine is the platform itself. There is no universally accepted platform at this time, with different institutions adopting different programs. Our study was the first to investigate what telemedicine platforms were most commonly being used among urologists and to compare the usability of these platforms based on a modified version of the TUQ. The 3 most commonly used telemedicine platforms by our participants were Zoom, Doxy.me, and Epic. There were no significant differences between the platforms in any category. Of note, of the top 5 most used platforms, Zoom, WhatsApp, and Skype are not designed for telemedicine despite being used for this purpose. Prior to COVID-19, all telemedicine platforms had to adhere to strict Health Insurance Portability and Accountability Act (HIPAA) specifications. With COVID-19 and the push for telemedicine, emergency provisions allowed for the usage of non-HIPAA compliant technologies like Facetime by Apple and WhatsApp. It is still recommended that when possible urologists utilize reliable and secure platforms that meet HIPAA standards [22]. Telemedicine is still fairly new, and although there are many upsides to its use, our data suggest that there is still much room for improvement for the technology to better accommodate current health care practices.

### Limitations and Conclusion

There are several limitations to our study. As this study was distributed partly through social media, participation may be skewed to urologists who are more comfortable with computer technologies and therefore are more likely to use telemedicine in their practice. Although we were able to capture data on over 600 urologists, it is only a small fraction of the entire urology community and therefore may not be an accurate representation of the community. It also must be noted that the majority of participants were from North America and Europe, which share similar Western socioeconomic qualities compared to the less represented urologists from Asia, South America, and Africa. Therefore, our study may be considered a more accurate representation of telemedicine usage in Western countries. In addition, our use of telemedicine as defined by video calls may have excluded a large portion of health care provided by urologists that was performed over the phone. Video calls may not be well established in many countries and the role of telephone consultations was not assessed in our study. Strategies to improve the responses in online survey studies include sending reminders to respond, offering incentives to respond, and keeping the surveys short. Despite these limitations, this is the first study to quantify the use, usability, and pervading interest in telemedicine among urologists during the COVID-19 pandemic. In the face of this pandemic, urologists have demonstrated the ability to adopt and adapt telemedicine into their practices, but barriers involving the technology itself are preventing many from utilizing it despite increasing interest.

## Abbreviations

ANOVA: analysis of variance
HIPAA: Health Insurance Portability and Accountability Act
IRB: institutional review board
TUQ: Telehealth Usability Questionnaire
